# 

**Published:** 1963-01

**Authors:** 


					vvn / ; wi h
January 1903
incorporating the medical journal of the south west
Vol 78 (i) N? 287
.<.v*'K 'j.* ., ?'*=' U ?z.f /
?

				

